# Insights into the Structural and Proteomic Changes in *Eimeria tenella* Unsporulated Oocysts Treated with Sodium Hypochlorite

**DOI:** 10.3390/ani16010067

**Published:** 2025-12-25

**Authors:** Liu-Shu Jia, Qing-Jie Wang, Shun-Hai Zhu, Qi-Ping Zhao, Yu Yu, Hong-Yu Han, Hui Dong

**Affiliations:** 1 College of Life Sciences, Jiangxi Normal University of Science and Technology, Fenglin Avenue 605, Nanchang 330013, China; jialiushu@126.com; 2 Key Laboratory of Animal Parasitology of Ministry of Agriculture, Shanghai Veterinary Research Institute, Chinese Academy of Agricultural Sciences, Shanghai 200241, China; wqj21006@163.com (Q.-J.W.); zhushunhai@shvri.ac.cn (S.-H.Z.); zqp@shvri.ac.cn (Q.-P.Z.); yuyuyu20231030@163.com (Y.Y.); hhysh@shvri.ac.cn (H.-Y.H.); 3 Shaanxi Provincial Center for Animal Disease Prevention and Control, Xi’an 710003, China

**Keywords:** sodium hypochlorite, *Eimeria tenella*, oocyst wall, label-free quantitative proteomics

## Abstract

Sodium hypochlorite (NaClO) is widely used to purify oocysts in laboratories. While previous studies have extensively examined its effects on oocyst viability, pathogenicity, and sporulation rate, the impact of NaClO treatment on proteomic profiles remains uncharacterized. Transmission electron microscopy was used in the present study to characterize structural changes in unsporulated oocyst walls of *Eimeria tenella* treated with NaClO. The results indicated that NaClO treatment destroyed the bilayer wall of unsporulated oocysts, stripping away the outer wall and making the inner layer thicker. Label-free quantitative proteomics was employed to identify differentially expressed proteins (DEPs) in NaClO-treated (Et-T) and untreated (Et-C) unsporulated oocysts. Among 2422 identified proteins, 1345 were differentially expressed, with 1210 upregulated and 134 downregulated in Et-T vs. Et-C. Functional analysis revealed that upregulated proteins are predominantly associated with oocyst wall biosynthesis and cellular stress responses, whereas downregulated proteins are involved in outer wall assembly and structural integrity. Notably, 12 proteins—including 9 hypothetical proteins, acid phosphatase, adenylate cyclase, and microneme protein 2—were exclusively detected in the Et-C, indicating their potential essentiality in outer wall formation. These findings reveal the structure and protein composition of the oocyst wall of *E. tenella*, supporting research on its biosynthesis and environmental resilience.

## Simple Summary

Sodium hypochlorite (NaClO) is widely used for oocyst purification, yet the effect of NaClO on oocyst proteomic changes has not been reported. This study investigated the structural and proteomic alterations in *Eimeria tenella* unsporulated oocysts induced by NaClO treatment. Transmission electron microscopy revealed that NaClO disrupts the oocyst wall bilayer by removing the outer layer and inducing thickening of the inner layer. Label-free quantitative proteomics identified 1344 differentially expressed proteins (DEPs) between the NaClO-treated (Et-T) and the untreated (Et-C) unsporulated oocysts. Functional analysis showed that DEPs are primarily associated with oocyst wall biosynthesis, stress response pathways, outer wall formation, and structural integrity. These findings provide critical insights into the molecular architecture of the oocyst wall and establish a foundation for elucidating the mechanisms underlying its biosynthesis and environmental resilience.

## 1. Introduction

Avian coccidiosis is a prevalent parasitic disease that results from infections by obligate intracellular parasites belonging to the *Eimeria* genus [1]. *Eimeria tenella* is recognized as the most pathogenic species associated with coccidiosis. This parasite has a specific site of parasitism, leading to the destruction of cecal/intestinal mucosa, as well as causing inflammation in the intestines and disintegration of epithelial cells [2]. This disease significantly impairs chicken growth and development, causing major economic losses exceeding £10.36 billion annually to the global poultry industry [3].

*E. tenella* undergoes a complex life cycle within the cecal epithelium of chickens following the ingestion of sporulated oocysts. This process involves three cycles of schizogony (asexual) followed by a subsequent phase of gametogony (sexual), culminating in the shedding of unsporulated oocysts with feces, which then proceed to undergo sporulation [4,5]. The unsporulated oocysts are the endpoint of sexual reproduction, possessing a robust bilayered wall that is fundamental to their environmental resilience. This structural barrier not only maintains oocyst integrity after excretion from the host but also effectively shields the internal undifferentiated protoplasm from a wide range of external physicochemical stressors. By protecting the parasite during its extracellular phase in the environment, this protective envelope ensures the successful completion of sporulation under permissive conditions and underpins the transmission potential and subsequent infection of new hosts [6,7]. To study the biological and molecular characteristics of oocysts, a large number of coccidial oocysts need to be isolated from *Eimeria*-infected chicken feces and sterilized and purified with sodium hypochlorite (NaClO). NaClO is widely used as a disinfectant on surfaces in settings such as healthcare facilities and food production plants, and is recognized as a cost-effective method for reducing the burden of waterborne diseases [8,9], and it also has the ability to disrupt biofilms and degrade structural proteins [10]. Many studies have successfully used NaClO solutions to clean fecal debris from oocysts of several species of coccidia [11,12,13]. However, when NaClO is used to treat oocysts, concentration and time must be strictly controlled. Studies have shown that some oocysts appear to break the oocysts wall and sporocysts overflow when treated with NaClO for more than 40 min and 5–10% NaClO solution can strip the outer wall of oocysts, cause damage to the structure of oocyst wall, and then affect the vitality of oocysts [14,15,16].

The effect of NaClO on the ultrastructure and protein profile of coccidian oocysts has not yet been reported. In this study, we systematically investigated structural changes in the wall of unsporulated oocysts of *Eimeria tenella* and alterations in protein expression following NaClO treatment by integrating transmission electron microscopy (TEM) with Label-free quantitative proteomics. The primary objective was to identify key functional proteins associated with outer oocyst wall formation and inner wall stabilization. Furthermore, quantitative real-time PCR (qPCR) was applied to validate transcriptional changes corresponding to the significantly differentially expressed proteins identified in the proteomic analysis. These findings offer novel insights and establish a theoretical foundation for understanding the protein composition, structural regulation, and biogenesis of the *E. tenella* oocyst wall.

## 2. Materials and Methods

### 2.1. Animals and Parasites

The protocol for the animal experiment received approval from the Animal Care and Use Committee at the Shanghai Veterinary Research Institute, Chinese Academy of Agricultural Sciences (Permit Number: SHVRI-SZ-20230323-4 on 23 March 2023). All procedures followed the approved guidelines to ensure adherence to ethical standards throughout the study.

One-day-old Three-yellow chickens were housed in temperature-controlled isolators (coccidia-free) with a constant ambient temperature of 30 ± 1 °C and a relative humidity of 50 ± 10%, and a 16 h:8 h light–dark cycle was maintained. Feed and water were provided ad libitum using a commercially available anticoccidial-free starter diet.

The *E. tenella* Shanghai strain was isolated from a commercial chicken farm in Shanghai and has been maintained in our laboratory since its original isolation [17]. The parasite was propagated through passage in coccidia-free chickens aged two weeks. Fecal samples were collected from chickens infected with *E. tenella* at 6–8 days post-infection. The fecal material was sequentially filtered through stainless steel sieves with mesh sizes of 80, 100, and 120 to collect the filtrate and centrifuged at 3200 rpm for 10 min. The pellet containing unsporulated oocysts (UO) was retained following supernatant removal.

### 2.2. Experimental Design

The omics experiment was divided into the NaClO-treated group (Et-T) and the control group (Et-C). The groups were treated as follows: (1) Et-T: 50% NaClO solution (with 4.5–5.0% available chlorine) was added to unsporulated oocysts, which were then repeatedly agitated on ice for 30 min and centrifuged at 2500 rpm for 10 min to extract the upper layer (i.e., oocysts) as previously described [18]. Subsequently, residual NaClO was removed by extensive washing with water. (2) Et-C: saturated salt flotation was used, according to Jenkins et al. (2017) [19], to obtain partially purified oocysts from fecal samples, serving as the control group. Previous studies have indicated that this treatment does not damage the wall of *Eimeria* oocysts [20].

Each group of samples was prepared in triplicate. All oocysts were washed thoroughly with sterile PBS and stored in liquid nitrogen.

### 2.3. Transmission Electron Microscopy

Unsporulated oocysts from the Et-C and Et-T groups were subjected to fixation with 1% glutaraldehyde at ambient temperature for an hour, followed by additional fixation using 1% osmium tetroxide for half an hour. Subsequent to the final wash in buffer and ethanol dehydration, the fixed samples were cleared with propylene oxide and subsequently embedded in a 1:1 mixture of Epon and Araldite (Pelco International, Fresno, CA, USA). Ultrathin sections measuring 40–60 nm were created and stained using uranyl acetate (Sigma-Aldrich, St. Louis, MO, USA) and lead citrate (Aladdin, Shanghai, China). Ultimately, these sections were examined under a transmission electron microscope (CM10, Philips, Amsterdam, The Netherlands) [21].

### 2.4. Protein Extraction and Protein Digestion

Proteins were isolated from samples utilizing SDT lysis buffer composed of 4% SDS (Bio-Rad, Hercules, CA, USA), 100 mM DTT (Sigma-Aldrich, St. Louis, MO, USA), and 100 mM Tris-HCl at pH 8.0. The samples were homogenized by vortexing with an equal volume of glass beads for 3 to 5 min. Samples were boiled for 5 min, further ultrasonicated, and then boiled for another 5 min. Undissolved cellular debris was removed by centrifugation at 16,000× *g* for 15 min. For digestion, 200 μg of protein from each sample was processed using the FASP method as outlined by Wiśniewski et al. (2009) [22]. Briefly, the UA buffer was supplemented with detergent, DTT, and IAA (Sigma-Aldrich, St. Louis, MO, USA) to prevent the reduction of cysteine residues. Finally, the protein suspension was digested overnight at 37 °C with trypsin (Promega, Madison, WI, USA) at a 50:1 ratio. Peptides were then collected by centrifugation at 16,000× *g* for 15 min and desalted using a C18 StageTip (Thermo Fisher Scientific, Waltham, MA, USA) before LC-MS (Thermo Fisher Scientific) analysis. The peptide concentrations were determined at an OD280 using a Nanodrop One device (Thermo Fisher Scientific, Waltham, MA, USA).

### 2.5. LC–MS/MS Analysis

The MS data were analyzed using MaxQuant software version 2.0.1.0 (MPI of Biochemistry, Martinsried, Germany). MS data were searched against the ToxoDB-59_EtenellaHoughton_AnnotatedProteins.fasta. An initial search was set with a precursor mass window of 6 ppm. The method involved an enzymatic cleavage approach utilizing trypsin KR/P, with a mass tolerance of 20 ppm for fragment ions; up to two missed cleavage sites were permitted. The results from the database search were filtered and subsequently exported, achieving a false discovery rate (FDR) of less than 1% at both the peptide-spectrum-matched level and the protein level. Label-free quantification was performed in MaxQuant, employing an intensity determination and normalization algorithm as outlined in prior studies [23,24,25]. The “LFQ intensity” for each protein across various samples was calculated to provide the most accurate estimate, fulfilling all pairwise peptide comparisons. This LFQ intensity closely matched the aggregated peptide intensities. Protein ratios were weighted and normalized using the median ratio in MaxQuant software. Only those proteins exhibiting a change of ≥1.5-fold along with a *p*-value of less than 0.05 were deemed to represent significant differential expressions.

### 2.6. Bioinformatics Analysis

Analyses of bioinformatics data were carried out using Perseus software (version 1.6.10.50) [26], Microsoft Excel, and R statistical computing software (version 3.5.3). Hierarchical clustering analysis was conducted utilizing the heatmap package, wherein the Euclidean distance served as the designated distance metric and complete linkage was employed as the chosen agglomeration method. Data was retrieved from sources including UniProtKB/Swiss-Prot [27], the Kyoto Encyclopedia of Genes and Genomes (KEGG) [28], and Gene Ontology (GO) [29] for the purpose of annotating the sequences. Enrichment analyses for GO and KEGG were performed using Fisher’s exact test, with false discovery rate (FDR) correction conducted to account for multiple testing. The GO terms were categorized into three groups: biological process (BP), molecular function (MF), and cellular component (CC) [30]. The enriched GO and KEGG pathways achieved nominal statistical significance at the *p* < 0.05 threshold.

### 2.7. Quantitative Real-Time PCR Assay

Ten genes were selected from the differently expressed unsporulated oocyst proteins to investigate transcription levels using qPCR [31]. Total RNA was extracted from unsporulated oocysts of Et-C and Et-T using TRIzol reagent according to the manufacturer’s instructions (Invitrogen, Thermo Fisher Scientific, Waltham, MA, USA) and reverse transcribed into cDNA using the HiScript III RT SuperMix for qPCR (+gDNA wiper) (Vazyme, Nanjing, China). qPCR analysis was performed on complementary DNA using the QuantiNova SYBR Green PCR Kit (Qiagen, Hilden, Germany) in combination with custom-designed primers. The primers, which were specific to the target genes, were synthesized by Sangon Biotechnology Co. (Shanghai, China) and are listed in Table 1. The 18S ribosomal RNA (rRNA) of *E. tenella* was simultaneously amplified as an internal reference gene [32]. The relative expression levels were determined using the 2^−ΔΔCt^ method [33], with each sample analyzed in three replicates.

### 2.8. Statistical Analysis

The relative gene expression levels in the Et-C and Et-T groups are presented as the mean ± standard deviation (SD). The statistical significance between samples was evaluated by performing Student’s *t*-test, with *p*-values less than 0.05 regarded as statistically significant, *p* < 0.01 indicated highly significant difference, *p* < 0.001 indicated extremely significant difference.

## 3. Results

### 3.1. Ultrastructural Changes in Oocyst Walls After Treatment with NaClO

**Figure 1:**
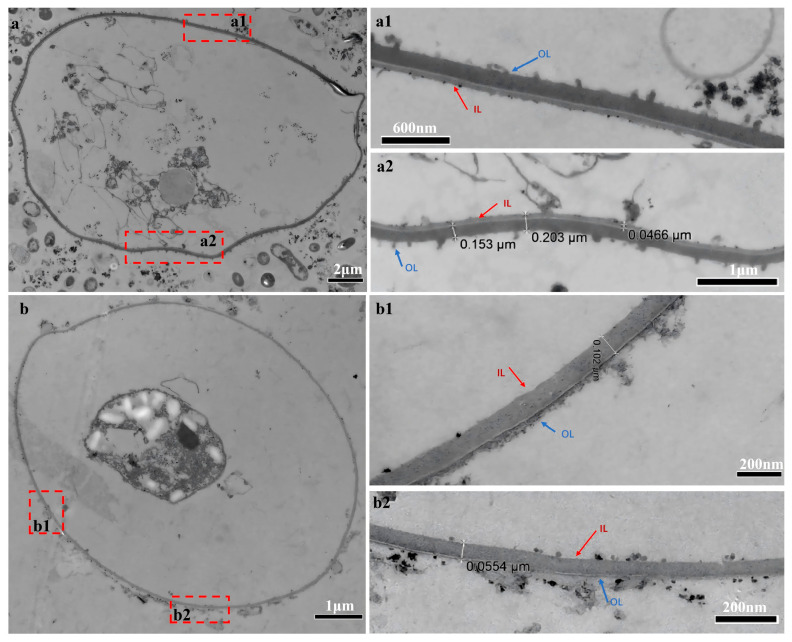
Transmission electron microscopy (TEM) illustrating the ultrastructural features of the oocyst wall of unsporulated oocysts. Panels (**a**), (**a1**,**a2**) depict oocysts from the control group (Et-C). Scale bar: 2 μm. Panel (**a**) shows unsporulated oocysts with an intact bi-layered wall structure; high-magnification views (**a1**,**a2**) reveal the thick, electron-dense outer layer (OL) and the thinner, more electron-lucent inner layer (IL) of the oocyst wall. Scale bars: 600 nm in (**a1**) and 1 μm in (**a2**). Panels (**b**), (**b1**,**b2**) show oocysts from the NaClO-treated group (Et-T). Panel (**b**) displays oocysts following NaClO treatment. Scale bar: 1 μm. Higher-magnification images (**b1**,**b2**) demonstrate partial removal of the outer wall and marked thickening of the inner layer. Scale bars: 200 nm in both (**b1**,**b2**).

Oocyst walls from control group remained an intact bilayer structure. The outer layer appeared electrodense with a roughened appearance. The outer surface had a thickness of 0.153 μm–0.161 μm. The electron-lucent inner layer appeared much thinner and had a thickness of 0.0466 μm–0.0784 μm (Figure 1(a1,a2)). However, in NaClO-treated samples, the bilayer structure of the oocyst wall was destroyed. The outer wall was stripped away; generally, only the inner layer was seen. The texture of the inner wall was loose, and the intermolecular gaps were increased. Its thickness was 0.0871 μm–0.102 μm. The inner wall was visually noticeably thickened (Figure 1(b1,b2)).

### 3.2. Proteins Detected in Unsporulated Oocysts of E. tenella

**Figure 2:**
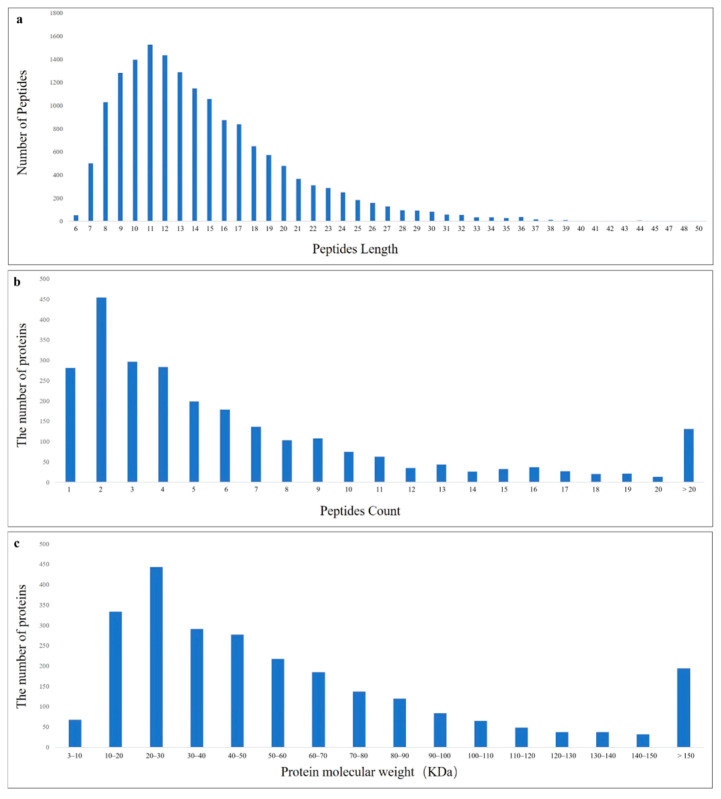
LC-MS/MS analysis of groups. (**a**) Peptides length distribution; (**b**) Proteins peptides identification number distribution; (**c**) Proteins molecular weight distribution.

A total of 16,401 peptides (Table S1) and 2422 proteins (Table S2) were identified in unsporulated oocysts of *E. tenella* using the label-free proteomic approach. The lengths of the peptides varied between 7 and 20 amino acids, with 95% of the identified peptides measuring under 32 amino acids (Figure 2a). Analysis of the unique peptide counts for the identified proteins revealed that the majority were characterized by 1 to 20 peptides, and most of these proteins had fewer than 10 peptide segments (Figure 2b). Regarding the distribution of protein masses, extensive coverage was achieved across a broad spectrum of molecular weights for proteins under 160 kD (Figure 2c).

**Figure 3:**
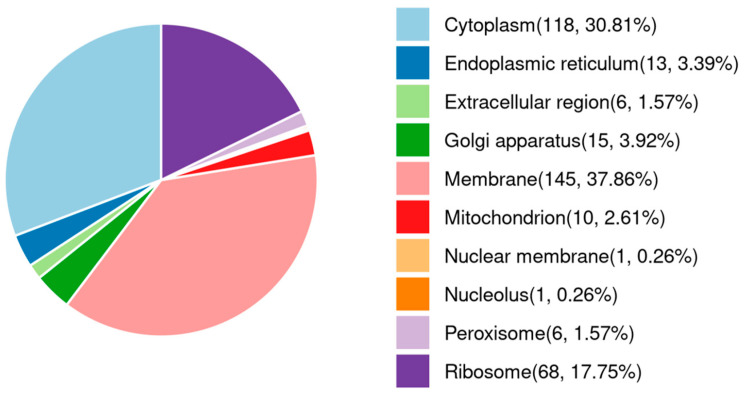
Predicted subcellular localization of unsporulated oocysts of *Eimeria tenella* proteins.

Of these proteins, 2389 co-existed in both the Et-T and Et-C groups, which grouped according to their subcellular locations as follows: 145 proteins were expressed in membranes (37.86%), 118 proteins were located at the cytoplasm (30.81%), and 68 proteins (17.75%) were expressed in ribosomes (Figure 3). The top 50 proteins with the most abundance in unsporulated oocysts of *E. tenella* are listed in Table 2. The 56 kDa gametocyte antigen, elongation factor 1-α, actin, equisetin synthetase, protein disulfide isomerase, heat shock protein 90, heat shock protein 70, glycogen phosphorylase family protein, fructose-bisphosphate aldolase, and enolase 2 were the top 10 most abundant proteins.

### 3.3. Differentially Expressed Proteins (DEPs) in NaClO-treated Unsporulated Oocysts of E. tenella

**Figure 4:**
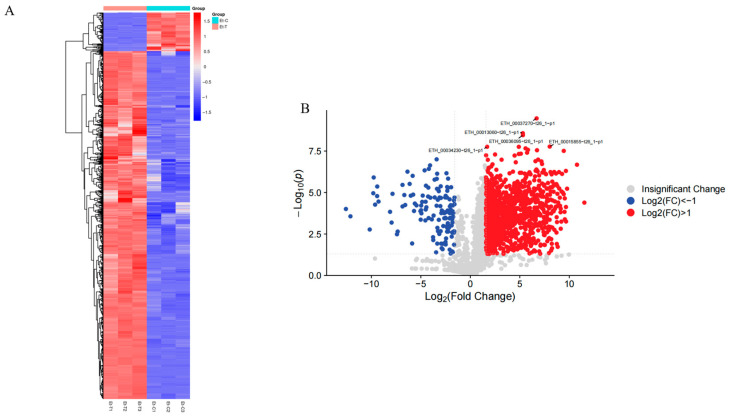
Hierarchical cluster analysis was conducted for all the differentially expressed proteins (DEPs) in Et-T and Et-C groups. (**A**) Heat map; (**B**) Volcano map.

Although identical total protein concentrations were utilized in samples from Et-T and Et-C unsporulated oocysts, we found considerable differences in individual protein levels between Et-T and Et-C (Table S3). The results from screening the DEPs have been visualized in hierarchical cluster analysis and volcano plots. Hierarchical cluster analysis was performed for all the DEPs for the Et-T vs. Et-C group (Figure 4A). The intra-group variation was minimal, and the inter-group comparability was high. In Figure 4B, upregulated protein expression is displayed in red, whereas downregulated expression is shown in blue. Compared with the untreated group, 1344 proteins displayed statistical changes in their expression levels (*p* < 0.05) in the NaClO-treated unsporulated oocysts, with 1210 upregulated and 134 downregulated. Additionally, 1045 proteins were not significantly changed between them.

The detailed upregulated data of 611 previously described proteins and 599 hypothetical proteins were observed in NaClO-treated unsporulated oocysts of *E. tenella*, of which 35 protein kinases, 17 proteasomes, 17 ubiquitins, 15 zinc finger proteins, 13 ribosomal proteins, and 6 RNA binding proteins displayed over a 500-fold change compared with the untreated unsporulated oocyst group (Table S4). Additionally, 16 proteins were associated with oocyst wall biosynthesis (Table 3), 15 proteins were related to the translation process of proteins (Table 4), 17 proteins were associated with response to stimuli of the *E. tenella* unsporulated oocysts (Table 5), and 25 proteins included splicing factors, DnaJ domain-containing proteins, and SAGs (Table 6).

The downregulated DEPs included 67 proteins that had been previously reported and 67 proteins with unknown functions. The top 35 proteins with the highest abundance in the outer wall of unsporulated oocysts are listed in Table 7. Histone H2A, tubulin beta chain, ATP synthase alpha chain, histone H4, tubulin alpha chain, and histone H2B were the most abundant proteins. Additionally, 18 proteins of interest were identified, such as microneme proteins, elongation factor G, and several enzymes (Table 8). Interestingly, 12 proteins specific for group Et-C were identified, including nine hypothetical proteins, acid phosphatase, adenylyl cyclase, and microneme protein 2 (Table 9).

### 3.4. GO Annotations and KEGG Pathway Analysis of DEPs

**Figure 5:**
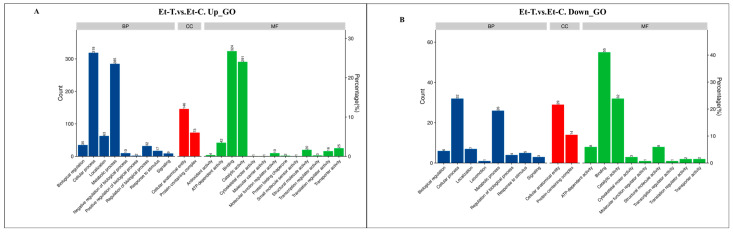
Gene ontology (GO) analyze of differentially expressed proteins (DEPs). Functional classification of up- (**A**) and down-regulated (**B**) proteins by GO analysis into categories of Biological Process (BF), Molecular Function (MF) and Cellular Component (CC).

To further understand the changes observed in unsporulated oocysts of *E. tenella* upon NaClO treatment, DEPs were categorized into three functional categories—biological process, molecular function, and cellular component—based on the Gene Ontology (GO) classification system and the UniProt database. In the 1210 upregulated DEPs, the most prevalent biological processes were cellular processes (319 proteins) and metabolic processes (285). The most prevalent cellular component was the cellular anatomical entity (146) and protein-containing complex (73). The predominant molecular functions were binding (324), catalytic activity (291), ATP-dependent activity (42), and transporter activity (25) (Figure 5A). Similarly, among the 134 downregulated DEPs, the dominant components of the biological processes and cellular components were the same as the upregulated DEPs. The most prevalent molecular functions included structural molecule activity (8) and binding, catalytic activity, and ATP-dependent activity (Figure 5B).

**Figure 6:**
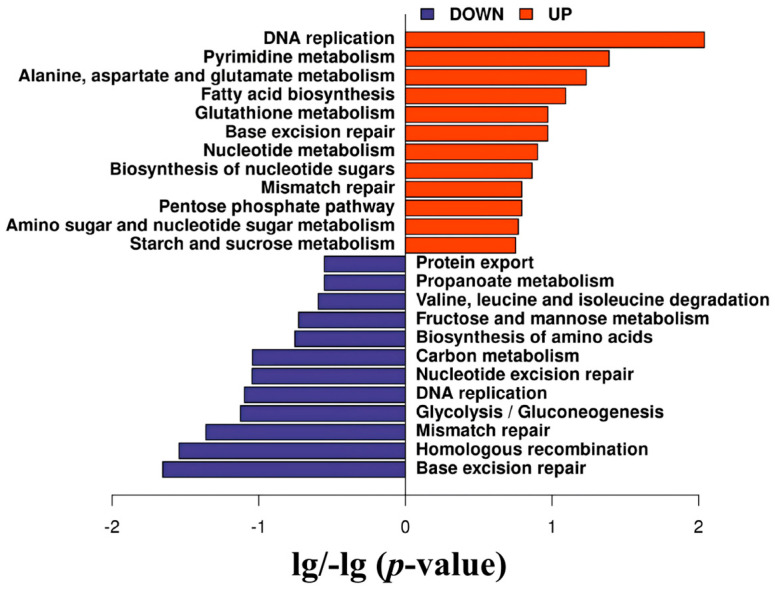
Kyoto Encyclopedia of Genes and Genomes (KEGG) analysis of the top 12 pathways with the most significant numbers of differentially expressed proteins (DEPs). The red represents the enrichment pathway of up-regulated DEPs. The blue represents the enrichment pathway of down-regulated DEPs.

The biological functions of these 1344 differentially expressed unsporulated oocyst proteins were further analyzed with the KEGG database, which mapped them to 40 pathways (Table S5). According to the *p*-value, Figure 6 presents the pathways with the top 12 enrichment significance. The upregulated DEPs were involved in DNA replication, pyrimidine metabolism, alanine-aspartate and glutamate metabolism, and fatty acid biosynthesis. In addition, the downregulated DEPs were associated with base excision repair, homologous recombination, mismatch repair, and glycolysis/gluconeogenesis.

### 3.5. Validation of Label-Free Proteomic Results with qPCR

Ten proteins were selected through qPCR to confirm the reliability of the proteomic results. These included four upregulated, three downregulated, and three non-significant.

**Figure 7:**
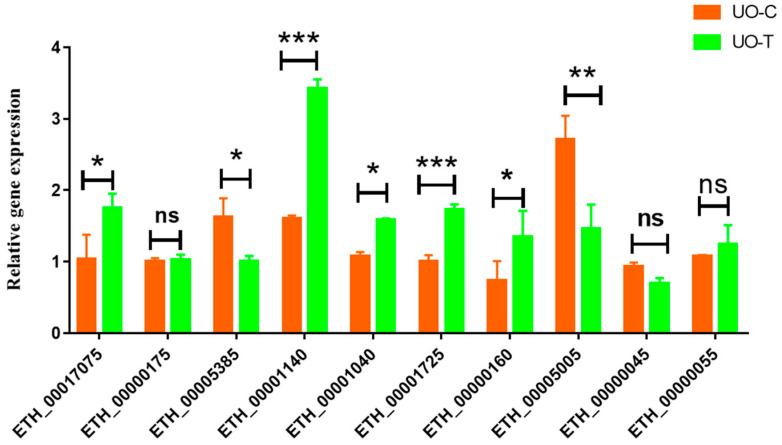
Assessment of gene expression by qPCR of ten genes selected from the label-free proteomic results. * represented significant difference (*p* < 0.05), ** indicated highly significant difference (*p* < 0.01), *** shown extremely significant difference (*p* < 0.001), ns was nonsignificant (*p* > 0.05).

The mRNA expression levels detected with qPCR were consistent with those obtained by proteomics for seven proteins, including acid phosphatase (ETH_00005385), AGC kinase (ETH_00001040), aspartyl proteinase (Eimepsin) (ETH_00001725), acetyltransferase domain-containing protein (ETH_00000160), prolyl-tRNA synthetase (ETH_00000045), and two hypothetical proteins (ETH_00000175 and ETH_00000055). There was 70% agreement between the qRT-PCR and proteomic results. Results for three proteins did not agree with the proteomic data: adenylyl cyclase (ETH_00017075), hypothetical protein (ETH_00001140), and ATP-binding cassette sub-family F member 1 (ETH_00005005) (Table 10 and Figure 7). These results indicate that most proteins were regulated directly at the transcriptional level. Nevertheless, there were instances where the levels of gene transcripts did not align with those of the respective proteins. This discrepancy may imply that protein abundance is not solely reliant on transcript levels but may also be influenced by post-translational modifications [34].

## 4. Discussion

Oocysts are the most resistant stage of the *Eimeria* life cycle. Oocysts may maintain their infectivity following exposure to disinfectants such as bleach, free chlorine, chlorine dioxide, and chloramine, when applied at concentrations and for durations commonly used in domestic or industrial settings [35,36,37]. In sporulated oocysts, the bilayered oocyst and sporocyst walls act as robust, almost hermetic barriers that shield the sporozoites from the harmful impacts of diverse environmental stressors, including both physical and chemical agents [38]. In unsporulated oocysts, the protoplasmic mass is protected by the double-layered oocyst wall, allowing for successful sporulation in a suitable environment. Structural damage to the oocyst wall, compromises the barrier function of the oocyst or sporocyst, leading to cytoplasmic leakage, premature excystation, or exposure of internal stages, which reduces infectivity. Oocysts can also activate compensatory mechanisms, including resistance from tyrosine-rich structural proteins, upregulation of stress-response [39] and redox pathways [40], and stage-specific resource reallocation for repair or development [41]. Although NaClO disrupts wall integrity and impairs viability [14,15], differences in stage susceptibility and intrinsic adaptation reveal the complexity of oocyst persistence in diverse environments.

According to the TEM results of the present study, the non-treated oocysts in the Et-C group retained their typical double-layered wall (observed thickness ~200 nm); that is, the inner layer (observed thickness ~60 nm) and the outer layer (observed thickness ~150 nm). In contrast, the outer layer was absent when oocysts were treated with 50% NaClO on ice for 30 min, with only the inner layer (observed thickness ~90 nm) remaining. In some instances, slight remnants of the outer layer persisted. The oocyst wall thickness was consistent with those reported in the literature (90 nm for the inner layer) [42]. After treatment with NaClO, it was observed that the inner layer was thickened and had become looser and sparser. This phenomenon may be attributed to the absent outer layer, leading to enlarged intermolecular gaps within the inner layer [43]. Ultrastructural changes in the oocyst wall may alter the composition of proteins.

Previous studies have demonstrated that both the functional activity and expression levels of proteins exhibit variability under different experimental conditions, such as alterations in temperature, nutrient availability, oxidative stress, hypoxic environments, and exposure to various pharmacological agents or toxic compounds [44,45,46]. The rapid changes in the protein profile observed following sodium hypochlorite treatment are unlikely to result from de novo protein synthesis, but instead likely arise from oxidative modifications of existing proteins, altered protein solubility and extraction efficiency due to disruption of the oocyst wall structure, and the physical loss of proteins associated with the degraded outer oocyst layers. These findings suggest that the parasite may modulate protein expression as a response to external stress [47,48,49,50]. In this study, NaClO treatment upregulated 17 proteins, including DnaJ domain-containing protein (ETH_00006810), 3,5-cyclic-nucleotide phosphodiesterase (ETH_00011905), phosphatidylinositol 3-kinase (ETH_00030035), and serine/threonine protein phosphatase (ETH_00043830). The upregulated proteins likely contribute to oocyst wall integrity and stress adaptation. The DnaJ domain-containing protein may refold or stabilize oxidative stress-damaged wall-associated proteins [51]. 3′,5′-cyclic-nucleotide phosphodiesterase may regulate intracellular signaling, influencing cytoskeletal reorganization or membrane remodeling needed for structural maintenance [52,53]. Phosphatidylinositol 3-kinase supports membrane trafficking and lipid signaling [54], potentially aiding repair of the inner oocyst wall [55]. Serine/threonine protein kinase may phosphorylate key structural or regulatory proteins, enabling rapid post-translational responses after wall damage [56]. Additionally, 16 proteins involved in the biosynthesis of the oocyst wall, 15 proteins involved in protein transcription and translation, and some functional proteins were identified. These proteins showed similar identification results in the proteomics of *E. tenella* oocyst wall and *Toxoplasma gondii* oocyst wall [14,57]. The upregulation of these functional proteins may indicate a compensatory response to the downregulation of proteins related to the stability of the oocyst wall structure, or it may potentially signify one of the adaptive responses of parasites to external stimuli [43,58,59]. Furthermore, we found two types of proteins—cathepsin L-like thiol proteinase (ETH_00033530) and OTU-like cysteine protease (ETH_00040555)—to be involved in the synthesis of the oocyst wall protein and may participate in the sporulation process of unsporulated oocysts [60,61].

Research has shown that NaClO can effectively remove the outer layer of the oocyst [43,62], resulting in the downregulation or disappearance of specific proteins. In this study, the downregulated DEPs mainly included histones, ATP synthase, phosphoglycerate kinase, PAN domain-containing protein, GPI transamidase subunit PIG-U, and glycerol-3-phosphate dehydrogenase. Additionally, functional proteins such as 3-hydroxyisobutyryl-CoA hydrolase, microneme protein MIC4, fatty acid hydroxylase, and elongator complex protein 3 were also identified. These proteins were also identified in the proteomic analyses of sporozoites and merozoites [63]. These findings suggest that these proteins may play a role in maintaining the structural integrity of the oocyst wall, as well as contributing to the biological processes associated with parasite growth, survival, and virulence [64,65,66,67]. Interestingly, in the present study identified 12 proteins specifically found in the Et-C group, including nine hypothetical proteins, acid phosphatase (ETH_00005385), adenylate cyclase (ETH_00017075), and microneme protein 2 (ETH_00026625). This may be related to the removal of the outer wall. Three known proteins have been previously documented to localize on the cell membrane or participate in its biogenesis [49,68,69,70]. However, the functions of the nine hypothetical proteins remain unknown, and further in-depth studies are needed to reveal their potential mechanisms in maintaining the structural stability of the oocyst outer wall and in the process of biosynthesis.

## 5. Conclusions

This study represents the first report of the abundance and differences in protein composition of unsporulated oocysts of *E. tenella* before and after household bleach (NaClO) treatment. The DEPs identified may have pivotal implications for the survival of *E. tenella* oocysts, as well as for wall formation. Our findings validate the resilience of the inner oocyst wall to household bleach [37] and underscore its unforeseen role as a protective barrier. Additionally, 12 specific proteins were discerned in the Et-C group, holding significant promise for early-stage oocyst formation and biosynthesis research on oocyst inner/outer walls. These findings contribute to a deeper understanding of the molecular architecture and stress response mechanisms in *Eimeria* spp. oocysts. The identified DEPs, particularly those associated with oocyst wall integrity and oxidative stress response, provide valuable insights that may guide the development of novel anti-coccidial therapeutics or targeted disinfection approaches aimed at interrupting parasite transmission. It should be acknowledged, however, that the present study was restricted to unsporulated oocysts; the responses of sporulated oocysts—the infectious stage—may differ due to their unique structural and metabolic properties. Moreover, while Label-free proteomics enables a comprehensive characterization of the proteome, further functional validation is required to definitively elucidate the biological roles of candidate proteins in oocyst wall formation and resistance to NaClO treatment. Future studies incorporating stage-specific comparative analyses and functional assays will be essential to fully unravel the mechanisms underlying oocyst environmental persistence.

## Figures and Tables

**Table 1:** Primers used in the quantitative real-time PCR assays.

Protein.IDs	Fasta.Headers	Primer Sequence (5′-3′)
ETH_00017075	adenylyl cyclase	F: GGCTTAGGCTACGCAGAAGGAG R: CGTGTCTGAGAGGAAGCGGAAG
ETH_00000175	hypothetical protein	F: CTATTGCTTCTGCTGCTGCTGTG R: ACTCCTGCTGCCGCCATTTC
ETH_00005385	acid phosphatase	F: ACGAAGTGGGACGAAATGTTTGAAG R: GAGCCATCGCCGTACACCAG
ETH_00001140	hypothetical protein	F: CAGCAGCAGCAACAGCAACAG R: GCAGCAAACGCCGACTCAAG
ETH_00001040	AGC kinase	F: ACAGCACGAGCAGCAGCAG R: CAGCAGATAGAGCGGCACTAGC
ETH_00001725	Aspartyl proteinase (Eimepsin)	F: ATGCGTTCCCTTCTGGTCGTG R: CATCTTCGGGCTCTTCAAGTGTTTC
ETH_00000160	acetyltransferase domain-containing protein	F: CAGCAACAACTAACAGAAGCAGACC R: AGCAGCGAAGCAGCAGAAGG
ETH_00005005	ATP-binding cassette sub-family F member 1	F: GCAACAGCAGCCATGATTCTCAG R: CAACAGCAACACATCGGCATCC
ETH_00000045	prolyl-tRNA synthetase	F: TCGTGGCTGTTCAGGCAGTTATC R: GCACTTCGCAACAATCTCCATCC
ETH_00000055	hypothetical protein	F: TACAGTCGCCGCATAGCCAAC R: ACATCGTCGTCGTCTCTAAGGTAAC

**Table 2:** The 50 most abundant proteins in the unsporulated oocysts of *E. tenella*.

Protein.IDs	Protein Name	Unique.Peptides	Sequence.Coverage (%)	MW (kDa)	Score	Intensity	*t*-Test *p* Value
ETH_00007320	56 kDa gametocyte antigen	15	33.8	53.74	246.47	1.45 × 10^9^	0.004013
ETH_00010290	Elongation factor 1-alpha	24	59.3	49.101	323.31	5.78 × 10^8^	0.004013
ETH_00009555	actin	24	79.3	42.027	263.26	5.47 × 10^8^	0.001131
ETH_00015480	Equisetin synthetase	273	40.8	1028.1	323.31	4.94 × 10^8^	3.83 × 10^−6^
ETH_00006210	protein disulfide isomerase	22	58.8	53.042	223.41	4.82 × 10^8^	0.000465
ETH_00000210	hypothetical protein	28	47.2	79.892	242.23	4.74 × 10^8^	9.34 × 10^−5^
ETH_00007385	heat shock protein 90	33	48.1	82.498	323.31	4.5 × 10^8^	0.000849
ETH_00025545	heat shock protein 70	32	54	73.788	323.31	4.5 × 10^8^	0.005804
ETH_00024080	glycogen phosphorylase family protein	43	58.3	111.63	323.31	4.44 × 10^8^	2.96 × 10^−7^
ETH_00008600	Fructose-bisphosphate aldolase	19	63.9	38.812	218.37	4.13 × 10^8^	2.74 × 10^−5^
ETH_00024910	enolase 2	24	47	53.657	269.69	4.08 × 10^8^	8.31 × 10^−5^
ETH_00008865	glyceraldehyde-3-phosphate dehydrogenase	23	74	36.383	181.62	3.54 × 10^8^	3.11 × 10^−5^
ETH_00011145	pyruvate kinase	21	53.3	57.59	242.94	3.11 × 10^8^	0.000244
ETH_00015895	elongation factor 2	37	50.5	92.166	316.37	2.96 × 10^8^	1.08 × 10^−5^
ETH_00015140	phosphoglycerate kinase	25	74.8	42.333	257.5	2.74 × 10^8^	3.94 × 10^−5^
ETH_00007980	14-3-3 protein	21	74.7	31.66	312.5	2.73 × 10^8^	0.000859
ETH_00020250	p97 protein	40	59.4	92.543	323.31	2.59 × 10^8^	1.68 × 10^−6^
ETH_00020595	18 kDa cyclophilin	11	71.4	20.467	227.36	2.26 × 10^8^	1.62 × 10^−6^
ETH_00021285	carbonyl reductase	20	75.9	29.014	169.5	2.15 × 10^8^	2.41 × 10^−5^
ETH_00003915	phosphofructokinase	37	46.8	122.77	323.31	2.07 × 10^8^	6.37 × 10^−5^
ETH_00007315	hypothetical protein	13	35.3	59.051	112.55	1.88 × 10^8^	3.63 × 10^−5^
ETH_00015095	alanine dehydrogenase	16	44.5	43.854	189.89	1.84 × 10^8^	0.000337
ETH_00020315	actin depolymerizing factor	12	87.3	13.18	151.59	1.79 × 10^8^	1.91 × 10^−5^
ETH_00012380	cytosol aminopeptidase	31	75.2	56.305	228.57	1.74 × 10^8^	3.2 × 10^−7^
ETH_00011110	20 kDa cyclophilin precursor	8	28.8	32.416	58.886	1.58 × 10^8^	7.68 × 10^−5^
ETH_00029250	penicillin amidase domain-containing protein	27	33.2	102.61	274.85	1.57 × 10^8^	0.00019
ETH_00028385	amiloride-sensitive amine oxidase	51	39.5	183.04	323.31	1.57 × 10^8^	7.52 × 10^−5^
ETH_00005300	lactate dehydrogenase	18	57.7	39.428	165.54	1.5 × 10^8^	0.007149
ETH_00023355	hypothetical protein	29	52.2	78.611	323.31	1.34 × 10^8^	2.78 × 10^−5^
ETH_00012285	hypothetical protein	41	60.5	90.02	323.31	1.27 × 10^8^	4.8 × 10^−6^
ETH_00005865	nucleoside diphosphate kinase	11	84.3	17.7	84.056	1.11 × 10^8^	9.09 × 10^−6^
ETH_00031660	hypothetical protein	8	63.8	20.515	103.51	1.09 × 10^8^	0.000159
ETH_00040515	ATP-citrate synthase	24	30.7	92.823	176.42	1.07 × 10^8^	6.82 × 10^−6^
ETH_00009955	hexokinase	21	32.4	78.23	136.02	1.06 × 10^8^	1.39 × 10^−5^
ETH_00009450	membrane-associated calcum-binding protein	15	63.5	33.482	295.05	1.03 × 10^8^	8.09 × 10^−6^
ETH_00003570	cysteine proteinase	22	58.4	56.94	273.18	1.03 × 10^8^	6.02 × 10^−5^
ETH_00010025	transketolase	25	45.4	75.994	180.83	1.03 × 10^8^	1.09 × 10^−5^
ETH_00008315	tudor/staphylococcal nuclease domain-containing protein	29	43.5	100.06	279.26	1.01 × 10^8^	3.71 × 10^−6^
ETH_00023750	porin	17	69.5	31.504	165.76	9.9725 × 10^7^	0.973556
ETH_00006230	hypothetical protein	12	36.1	51.371	243.02	9.9126 × 10^7^	0.001652
ETH_00002575	thioredoxin	19	42.9	49.745	133.85	9.9005 × 10^7^	2.25 × 10^−5^
ETH_00008560	intracellular protease	18	12.7	191.11	151.57	9.872 × 10^7^	0.001779
ETH_00011830	Superoxide dismutase	8	53.7	23.72	179.72	9.8282 × 10^7^	7.17 × 10^−6^
ETH_00010625	heat shock protein	28	58.6	80.13	228.59	9.7681 × 10^7^	3.52 × 10^−6^
ETH_00004800	histone H2A	2	16.4	16.422	18.274	9.7542 × 10^7^	0.011746
ETH_00014435	sulfate adenylyltransferas-adenylylsulfate kinase	24	46	74.507	239.44	9.7541 × 10^7^	3.93 × 10^−6^
ETH_00021420	heat shock protein	40	52.8	104.11	323.31	9.7423 × 10^7^	8.29 × 10^−6^
ETH_00030905	triosephosphate isomerase	12	66.1	27.36	115.39	9.4539 × 10^7^	0.024076
ETH_00026340	heat shock protein 28	9	22.3	65.071	186.27	9.12 × 10^7^	0.000287
ETH_00030365	KH domain-containing protein	37	47.7	103.07	323.31	9.1069 × 10^7^	7.41 × 10^−7^

**Table 3:** Up-regulated DEPs proteins involved in the biosynthesis of the oocyst walls.

Gene IDs	Protein Description	Average Abundance	*t*-Test *p* Value	Fdr	Up/Down
ETH_00001980	peroxisome biogenesis factor 7	124,683.5	0.001443	0.002709	Up
ETH_00033530	cathepsin L-like thiolproteinase	659,375.8628	1.89216 × 10^−5^	0.000124872	Up
ET0H_00018950	peroxisomal membrane protein	15,603.4	0.00016	0.0005	Up
ETH_00027740	peroxisomal multifunctional enzyme type 2	9,492,651	6.83 × 10^−6^	7.96 × 10^−5^	Up
ETH_00029535	peroxisome biogenesis factor 1	15,271.25	2.89 × 10^−5^	0.000159	Up
ETH_00032305	peroxidoxin 2	14,073,206	0.000169	0.000521	Up
ETH_00002595	3-oxoacyl-(acyl-carrier-protein) synthase III family protein	358,205	3.95 × 10^−6^	6.41 × 10^−5^	Up
ETH_00019675	very long-chain acyl-CoA synthetase	8,951,830	6.73 × 10^−6^	7.96 × 10^−5^	Up
ETH_00011645	long-chain fatty acid CoA ligase	3,100,979	8.9 × 10^−6^	8.7 × 10^−5^	Up
ETH_00000185	enoyl-acyl carrier reductase	6,914,303	1.27 × 10^−5^	0.000105	Up
ETH_00032045	acyl-coenzyme A oxidase	864,156.3	7.44 × 10^−5^	0.000295	Up
ETH_00040555	OTU-like cysteine protease domain-containing protein	118,260.3	4.15168 × 10^−6^	6.55465 × 10^−5^	Up
ETH_00027910	3-oxoacyl-[acyl-carrier-protein] synthase	1,200,924	3.75 × 10^−6^	6.41 × 10^−5^	Up
ETH_00002550	oxidoreductase	977,874.4	0.010412	0.01527	Up
ETH_00015230	oxidoreductase	341,557.7	2.66 × 10^−5^	0.000152	Up
ETH_00043030	quinone oxidoreductase	355,123.3	6.8 × 10^−5^	0.000276	Up

**Table 4:** Up-regulated DEPs proteins related to the translation process of proteins.

Gene IDs	Protein Description	Average Abundance	*t*-Test *p* Value	Fdr	Up/Down
ETH_00008900	eukaryotic translation initiation factor 5	306,508	0.003777	0.006254	Up
ETH_00015170	eukaryotic translation initiation factor 6	586,371.9	0.001091	0.002163	Up
ETH_00024135	eukaryotic translation initiation factor 4e	2,280,139	4.95 × 10^−5^	0.000222	Up
ETH_00024775	eukaryotic translation initiation factor 3 subunit g	1,962,599	0.00017	0.000523	Up
ETH_00027675	Eukaryotic translation initiation factor 3 subunit 9	2,810,655	7.03 × 10^−6^	8 × 10^−5^	Up
ETH_00027685	eukaryotic translation initiation factor 3 subunit 7	2,291,947	0.000484	0.001138	Up
ETH_00029105	eukaryotic translation initiation factor 2A	1,817,609	0.001029	0.002064	Up
ETH_00031135	eukaryotic translation initiation factor 2 alpha subunit	4,834,312	0.000178	0.000536	Up
ETH_00038350	eukaryotic translation initiation factor 3 subunit 10	2,770,224	7.03 × 10^−6^	8 × 10^−5^	Up
ETH_00038355	Eukaryotic translation initiation factor 3 subunit 10	44,818.92	9.35 × 10^−7^	3.29 × 10^−5^	Up
ETH_00041970	eukaryotic translation initiation factor 2 gamma subunit	2,802,917	7.66 × 10^−5^	0.0003	Up
ETH_00018510	translation elongation factor Tu	252,953.9	0.003838	0.006333	Up
ETH_00029140	elongation factor 1	4,112,507	5.75 × 10^−6^	7.51 × 10^−5^	Up
ETH_00033040	elongation factor Tu GTP-binding domain-containing protein	73,512.22	1.18 × 10^−5^	0.000101	Up
ETH_00040100	elongation factor 1-alpha	32,789.6	2.05 × 10^−5^	0.00013	Up

**Table 5:** Up-regulated DEPs proteins involved in the response to stimulus of the *E. tenella* of unsporulated oocysts.

Gene IDs	Protein Description	*t*-Test *p* Value	Log2FC	Fdr	Up/Down
ETH_00001650	importin-alpha re-exporter	2.858 × 10^−5^	1.631607	0.000158	Up
ETH_00002130	mRNA polyadenylation-related protein	0.008058539	4.03413	0.0122	Up
ETH_00004095	DNA mismatch repair protein	0.034288813	2.028477	0.044959	Up
ETH_00006560	hypothetical protein	0.000205239	2.778015	0.000596	Up
ETH_00006810	DnaJ domain-containing protein	0.00015132	1.699637	0.000482	Up
ETH_00011905	3,5--cyclic-nucleotide phosphodiesterase	0.03064539	6.445682	0.040493	Up
ETH_00018240	RAB GDP dissociation inhibitor alpha	0.000295158	1.605594	0.000783	Up
ETH_00019375	sec7 domain-containing protein	0.000204155	1.720751	0.000594	Up
ETH_00019445	CutA1 divalent ion tolerance domain-containing protein	6.25 × 10^−5^	1.795391	0.000259	Up
ETH_00022395	endonuclease/exonuclease/phosphatase domain-containing protein	4.35 × 10^−6^	1.932506	6.58 × 10^−5^	Up
ETH_00028060	hypothetical protein	0.000116467	2.51882	0.000403	Up
ETH_00030035	phosphatidylinositol 3-kinase	0.004276524	3.038657	0.006903	Up
ETH_00031560	structural maintenance of chromosome domain-containing protein	0.005618193	2.017605	0.008824	Up
ETH_00034370	selR domain-containing protein	4.81 × 10^−5^	4.475181	0.000217	Up
ETH_00036640	alkyl sulfatase	0.001024167	1.910875	0.00206	Up
ETH_00037265	DNA mismatch repair protein mutS	0.020734578	2.127737	0.028419	Up
ETH_00043830	serine/threonine protein phosphatase	0.000758965	1.628867	0.001626	Up

**Table 6:** Other functionally interesting proteins identified in up-regulated DEPs.

Gene IDs	Protein Description	Average Abundance	*t*-Test *p* Value	Fdr	Up/Down
ETH_00008655	splicing factor 3B subunit 2	122,875.7	0.00044	0.00105	Up
ETH_00009760	splicing factor U2AF 65 kDa subunit	199,777.6	0.00738	0.01126	Up
ETH_00010955	splicing factor 3A subunit 2	49,272.8	0.01591	0.02232	Up
ETH_00017895	splicing factor	100,455.5	2.6 × 10^−5^	0.00015	Up
ETH_00021870	step II splicing factor slu7	22,798.66	0.0468	0.06002	Up
ETH_00022985	splicing factor 3b subunit 10	65,827.72	0.02699	0.036	Up
ETH_00023985	splicing factor	1,647,924	6.2 × 10^−5^	0.00026	Up
ETH_00024100	splicing factor 3B subunit 1	496,787.6	0.02534	0.03403	Up
ETH_00001145	DnaJ domain-containing protein	687,210.8	8.5 × 10^−6^	8.6 × 10^−5^	Up
ETH_00005200	DnaJ domain-containing protein	15,390.68	0.01375	0.01958	Up
ETH_00006810	DnaJ domain-containing protein	5,791,514	0.00015	0.00048	Up
ETH_00014270	DnaJ domain-containing protein	35,176.8	3.9 × 10^−5^	0.00019	Up
ETH_00016875	DnaJ domain-containing protein	362,587.9	0.00122	0.00237	Up
ETH_00018980	DnaJ domain-containing protein	2,880,571	0.00011	0.00039	Up
ETH_00028825	DnaJ domain-containing protein	110,563.8	0.00693	0.01066	Up
ETH_00034365	DnaJ domain-containing protein	1,044,757	3.9 × 10^−5^	0.00019	Up
ETH_00042870	DnaJ domain-containing protein	586,182.9	0.00024	0.00067	Up
ETH_00003730	SAG family member	185,582.3	0.00219	0.00389	Up
ETH_00010835	SAG family member	1,743,194	0.00342	0.00573	Up
ETH_00024330	SAG family member	3,816,199	6.5 × 10^−7^	2.9 × 10^−5^	Up
ETH_00034880	SAG family member	131,364.5	0.00088	0.00183	Up
ETH_00034900	SAG family member	113,318.4	0.01617	0.02261	Up
ETH_00034935	SAG family member	962,103.4	0.00394	0.00646	Up
ETH_00035010	SAG family member	1,341,516	0.00351	0.00586	Up
ETH_00035025	SAG family member	535,658.4	9.1 × 10^−6^	8.7 × 10^−5^	Up

**Table 7:** The top 35 proteins with highest abundance in the outer wall of unsporulated oocysts.

Gene IDs	Protein Description		*t*-Test *p* Value	Fdr	Up/Down
ETH_00004800	histone H2A	94,563,323.7	0.011746	0.017006	Down
ETH_00002520	tubulin beta chain	25,748,366.6	3 × 10^−5^	0.000162	Down
ETH_00005270	ATP synthase alpha chain	24,232,117.9	0.002156	0.003829	Down
ETH_00028080	histone H4	16,265,569.5	0.012313	0.01772	Down
ETH_00033310	tubulin alpha chain	18,241,886	1.68 × 10^−6^	4.45 × 10^−5^	Down
ETH_00028290	histone H2B	16,633,592.6	0.000132	0.000441	Down
ETH_00002570	ATP-binding cassette protein subfamily B member 2	7,097,327.17	0.000994	0.002017	Down
ETH_00027415	phosphoglycerate kinase	6,932,781.07	0.001045	0.002088	Down
ETH_00009225	endonuclease V	6,885,985.01	0.000179	0.000537	Down
ETH_00026205	PAN domain-containing protein	7,611,667.77	3.02 × 10^−6^	6.07 × 10^−5^	Down
ETH_00015540	Splicing factor Prp8	7,169,337.57	0.000292	0.000776	Down
ETH_00033600	GPI transamidase subunit PIG-U	7,284,422.37	7.88 × 10^−6^	8.48 × 10^−5^	Down
ETH_00003225	U2 snRNP auxiliary factor small subunit	5,357,745.28	0.000244	0.000676	Down
ETH_00033360	oxidoreductase	6,513,587.33	3.59 × 10^−5^	0.000182	Down
ETH_00014995	translation initiation factor 2 beta	4,818,694.97	0.013958	0.019848	Down
ETH_00022360	glycerol-3-phosphate dehydrogenase	4,655,180.42	0.000967	0.001982	Down
ETH_00007685	Whole genome shotgun assembly, reference scaffold old set, scaffold scaffold_9	5,469,729.62	2.12 × 10^−5^	0.000132	Down
ETH_00015505	fructose-1,6-bisphosphate aldolase	5,155,371.7	4.81 × 10^−6^	6.89 × 10^−5^	Down
ETH_00018005	serine/threonine protein phosphatase	4,969,595.51	6.85 × 10^−5^	0.000277	Down
ETH_00011330	SERPIN1 protein	4,716,701.88	1.58 × 10^−5^	0.000114	Down
ETH_00027460	PAN domain-containing protein	4,844,767.32	1.53 × 10^−5^	0.000113	Down
ETH_00042760	zinc carboxypeptidase	4,820,694.93	4.61 × 10^−5^	0.000211	Down
ETH_00001490	kinesin motor domain containing protein	4,457,628.73	5.65 × 10^−5^	0.000242	Down
ETH_00019085	kinesin heavy chain	3,725,719.86	1.98 × 10^−5^	0.000128	Down
ETH_00020795	lytB domain-containing protein	3,177,181.18	5.97 × 10^−6^	7.6 × 10^−5^	Down
ETH_00016635	histone H2A	3,009,108.27	2 × 10^−5^	0.000128	Down
ETH_00005040	RNA binding motif-containing protein	2,608,578.04	1.41 × 10^−5^	0.00011	Down
ETH_00036450	kelch motif domain-containing protein	2,595,967.87	5.96 × 10^−5^	0.000252	Down
ETH_00011955	chromodomain helicase DNA binding protein	2,539,536.48	3.66 × 10^−5^	0.000184	Down
ETH_00023060	CTP synthase	1,780,127.82	0.000121	0.000415	Down
ETH_00004950	mitochondrial import inner membrane translocase subunit tim17	1,890,304.31	0.000117	0.000404	Down
ETH_00026425	GJ18811	1,539,163.95	5.07 × 10^−6^	7.02 × 10^−5^	Down
ETH_00002255	ubiquitin-conjugating enzyme e2	1,427,748.9	6.11 × 10^−5^	0.000256	Down
ETH_00027215	WD-repeat protein	1,293,674.48	0.002131	0.003788	Down
ETH_00021555	signal recognition particle 54 kDa protein	1,553,016.25	0.002301	0.00405	Down

**Table 8:** Other functionally interesting proteins identified in down-regulated DEPs.

Gene IDs	Protein Description	Average Abundance	*t*-Test *p* Value	Fdr	Up/Down
ETH_00026545	DnaJ domain-containing protein	1,295,291.3	0.000106	0.000374	Down
ETH_00031315	pinA	1,124,459.6	2.32 × 10^−7^	2 × 10^−5^	Down
ETH_00027715	glycosylphosphatidylinositol anchor attachment 1 protein	1,122,458.7	0.000175	0.00053	Down
ETH_00034470	3-hydroxyisobutyryl-CoA hydrolase, mitochondrial precursor	783,832.56	2.05 × 10^−5^	0.00013	Down
ETH_00021655	Micronemal protein MIC4	863,147.76	0.00013	0.000434	Down
ETH_00031400	glucose-methanol-choline oxidoreductase	651,304.37	0.000409	0.000997	Down
ETH_00029865	activating signal cointegrator 1 complex subunit 3	423,035.22	0.004531	0.007269	Down
ETH_00021010	microneme protein	479,839.57	9.88 × 10^−7^	3.37 × 10^−5^	Down
ETH_00019285	fatty acid hydroxylase	278,638.98	0.001048	0.002091	Down
ETH_00015025	diacylglycerol kinase	266,810.78	0.000707	0.001543	Down
ETH_00034460	elongation factor G	178,237.82	0.000415	0.001007	Down
ETH_00013180	SAG family member (sag14)	262,567.67	0.022663	0.030762	Down
ETH_00031485	elongator complex protein 3	146,292.65	0.002282	0.004027	Down
ETH_00030975	Na+/H+ antiporter	95,571.378	0.00732	0.011188	Down
ETH_00034940	SAG family member (sag12)	83,331.758	0.000433	0.001037	Down
ETH_00042760	zinc carboxypeptidase	4,820,694.9	4.61 × 10^−5^	0.000211071	Down
ETH_00033360	oxidoreductase	170,505.3	3.59 × 10^−5^	0.060916941	Down

**Table 9:** Down-regulated DEPs with group-specific for Et-C.

Gene IDs	Protein Description	Et-C (Average Abundance)	*t*-Test *p* Value	Control Mean	Control SD	FC	Log2FC	Fdr	Up/Down
ETH_00003050	hypothetical protein	1,608,483	0.000272	1,608,483	231,159.6	0.000225	−12.1178	0.000737	Down
ETH_00005385	acid phosphatase	224,774.4	3.5 × 10^−5^	224,774.4	19,185.81	0.00161	−9.27869	0.00018	Down
ETH_00005690	hypothetical protein	100,281.9	0.000146	100,281.9	12,247.73	0.003609	−8.11428	0.000471	Down
ETH_00007070	hypothetical protein	389,101.4	0.094865	389,101.4	308,922	0.001254	−9.63972	1.15 × 10^−5^	Down
ETH_00013320	hypothetical protein	2,207,863	9.8 × 10^−5^	2,207,863	244,735.4	0.000164	−12.5748	0.000356	Down
ETH_00014050	hypothetical protein	58,787.5	0.002247	58,787.5	14,552.78	0.006156	−7.3438	0.003979	Down
ETH_00016610	hypothetical protein	247,946.4	1.64 × 10^−5^	247,946.4	17,481.94	0.00146	−9.42025	0.000116	Down
ETH_00017075	adenylyl cyclase	90,000.9	0.000646	90,000.9	16,096.71	0.004021	−7.95823	0.001428	Down
ETH_00017995	hypothetical protein	416,678.7	0.00169	416,678.7	96,135.98	0.000869	−10.1692	0.003109	Down
ETH_00026625	microneme protein 2	315,343.8	1.24 × 10^−6^	315,343.8	11,639.9	0.001148	−9.76714	3.91 × 10^−5^	Down
ETH_00028660	hypothetical protein	245,961.9	4.35 × 10^−6^	245,961.9	12,430.88	0.001471	−9.40865	6.58 × 10^−5^	Down
ETH_00042300	hypothetical protein	5,243,104	1.1 × 10^−5^	5,243,104	334,707.3	0.001114	−9.81022	9.52 × 10^−5^	Down

**Table 10:** qPCR Verification of the proteomic data.

Gene IDs	Protein Description	Up/Down	qPCR-Up/Down
ETH_00017075	adenylylcyclase	Down	Up
ETH_00000175	hypothetical protein	NoSig	NoSig
ETH_00005385	acidphosphatase	Down	Down
ETH_00001140	hypothetical protein	Down	Up
ETH_00001040	AGC kinase	Up	Up
ETH_00001725	Aspartyl proteinase (Eimepsin)	Up	Up
ETH_00000160	acetyltransferase domain-containing protein	Up	Up
ETH_00005005	ATP-binding cassette sub-family F member 1	Up	Down
ETH_00000045	prolyl-tRNA synthetase	NoSig	NoSig
ETH_00000055	hypothetical protein	NoSig	NoSig

Note: NoSig, nonsignificant.

## Data Availability

Data are contained within the article and Supplementary Materials.

## Supplementary Materials

The following supporting information can be downloaded at: https://www.mdpi.com/article/10.3390/ani16010067/s1, Figure S1: Principal component analysis (PCA) score plot showing the overall proteomic differences in *Eimeria tenella* unsporulated oocysts in Et-T and Et-C groups; Table S1: Complete dataset of *E. tenella* unsporulated oocysts peptides identified in this study; Table S2: Complete dataset of *E. tenella* unsporulated oocysts proteins identified in this study; Table S3: All differentially expressed proteins between Et-T and Et-C in this study; Table S4: All known proteins in up-regulated DEPs; Table S5: KEGG database analysis of all DEPs.
