# Supplementary material for: Insights into the Structural and Proteomic Changes in Eimeria tenella Unsporulated Oocysts Treated with Sodium Hypochlorite
Source: Animals (Basel). 2025 Dec 25;16(1):67. doi: 10.3390/ani16010067 (PMC12784848; doi:10.3390/ani16010067)
Supplement: Supplementary file 1 [file animals-16-00067-s001.zip › Figure S1.pdf]

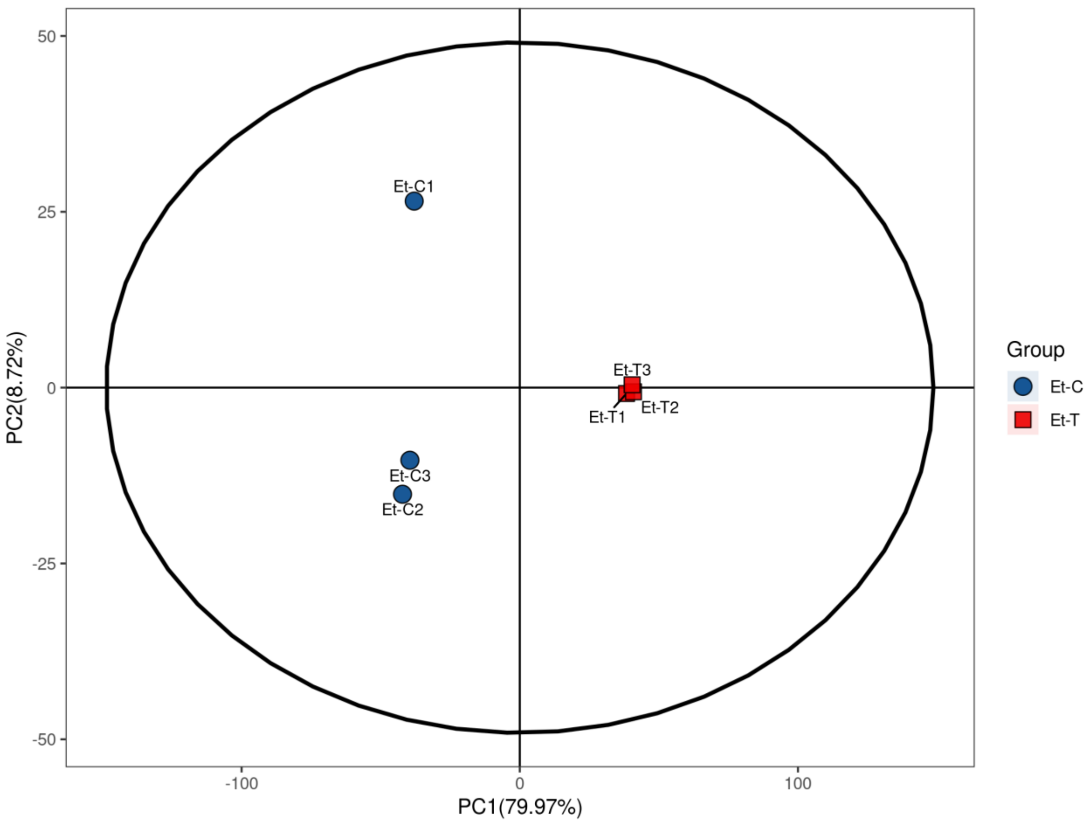

Figure S1: Principal component analysis (PCA) score plot showing the overall proteomic differences in *Eimeria tenella* unsporulated oocysts in in Et-T and Et-C groups.

The horizontal axis (PC1) and vertical axis (PC2) account for 79.97% and 8.72% of the total variance, respectively. Blue circles represent the untreated control group (Et-C, n = 3), and red squares represent the sodium hypochlorite-treated group (Et-T, n = 3). The distinct separation between the two groups along the PC1 axis indicates a significant treatment-induced alteration in the protein expression profile of the oocysts. Furthermore, the tight clustering of replicates within the Et-T group reflects high experimental reproducibility, whereas the more dispersed distribution of the Et-C group samples suggests the presence of inherent biological variability or technical variation.
